# Does urinary metabolite signature act as a biomarker of post-stroke depression?

**DOI:** 10.3389/fpsyt.2022.928076

**Published:** 2022-08-24

**Authors:** Wa Cai, Xia-Fei Wang, Xi-Fang Wei, Jing-Ruo Zhang, Chen Hu, Wen Ma, Wei-Dong Shen

**Affiliations:** ^1^Department of Acupuncture, Shanghai Shuguang Hospital Affiliated to Shanghai University of Traditional Chinese Medicine, Shanghai, China; ^2^Department of Neurology, Seventh People’s Hospital of Shanghai University of Traditional Chinese Medicine, Shanghai, China

**Keywords:** metabolomics, urinary metabolite, biomarker, post stroke depression, systematic review

## Abstract

**Background:**

It is difficult to conduct the precise diagnosis of post-stroke depression (PSD) in clinical practice due to the complex psychopathology of depressive disorder. Several studies showed that gas chromatography–mass spectrometry (GC-MS)-identified urinary metabolite biomarkers could significantly discriminate PSD from stroke survivors.

**Methods:**

A systematic review was performed for the keywords of “urinary metabolite” and “PSD” using Medline, Cochrane Library, Embase, Web of Science, PsycINFO, Wanfang, CNKI, CBM, and VIP database from inception to 31 March 2022.

**Results:**

Four related studies were included in the review. Differential urinary metabolites including lactic acid, palmitic acid, azelaic acid, and tyrosine were identified in all the included studies. As a significant deviation in the metabolite biomarker panel, glyceric acid, azelaic acid, phenylalanine, palmitic acid, pseudouridine, and tyrosine were found in at least 2 included studies, which indicated good potential for the differentiation of PSD.

**Conclusion:**

The systematic review provided evidence that differential urinary metabolites analyzed by the GC-MS-based approach might be used as a biomarker for the diagnosis and prognosis of PSD.

## Highlights

- First systematic review was conducted to investigate the association between urinary metabolite and PSD.
- All the included studies identified differential urinary metabolites including lactic acid, azelaic acid, tyrosine, and palmitic acid.

- Azelaic acid, glyceric acid, palmitic acid, phenylalanine, pseudouridine, and tyrosine were found significantly differentiated in the metabolite biomarker panel, indicating good potential for the differentiation of PSD.
- The systematic review provided evidence that differential urinary metabolites analyzed by GC-MS-based might be used as a biomarker for the diagnosis and prognosis of PSD.

## Introduction

Stroke is a serious cerebrovascular disease with high disability and mortality. Several complications were found in stroke survivors, including depression (1), anxiety (2), fatigue (3, 4), apathy (5), insomnia (6), psychosis (7), mania (8), dementia (9), cognitive disorder (10), and anosognosia (11). Of these, depression after stroke was studied by most researchers due to its high prevalence rate. It was indicated in a meta-analysis including 61 studies enrolling 25,488 patients that the morbidity of depression in stroke survivors was 31% (12). A number of representative studies (13–23) reported that the incidence rate of post-stroke depression (PSD) is from 11 to 41%, negatively impacting the life quality of patients with stroke and imposing heavy economic burdens on their families. Referring to previous studies, prior history of depression or severe stroke is significant risk of PSD (24–27), whereas marriage (28), social support (29, 30), and levels of education (31) are ambiguous risk factors. However, the pathophysiological mechanisms of PSD are complex and remain to be clarified, which were found to be related to hypothalamic-pituitary-adrenal (HPA) axis dysfunction (32, 33), increased inflammation (34, 35), changes in neurotransmitters (36), and decreased neurotrophic factors (37–39).

Although the depressive disorder can be diagnosed by classical clinical nosologies (40), the diagnosis fundamentals of depression were under more disagreements (41, 42). Symptoms of depression were found to be a result of phenotypic, biological, genetic heterogeneity, and complex interactions between biological and environmental factors. Therefore, not fully understanding the complex psychopathology of depression leads to an ineffective treatment strategy (43). Fortunately, metabolomics is a quantifiable monitor of biochemical state to inform the molecular mechanisms of disorders. Hence, metabolomics acted as diagnostic, prognostic biomarkers (44) for psychiatric disorders in numerous studies. A urinary metabolomics study indicated that two sets of metabolites that could effectively discriminate “moderate” and “severe” patients with depression from healthy controls (HCs) were identified by nuclear magnetic resonance (NMR)- and gas chromatography–mass spectrometry (GC-MS)-based methods (45). Another study found that several specified metabolites could act as predictors of depression recovery (46, 47). Of note, recent studies (48–51) showed that GC-MS-identified urinary metabolite biomarkers could significantly distinguish PSD from stroke survivors, which could be used as an objective diagnostic tool for PSD.

The systematic review was conducted to clarify the specific role of urinary metabolites in PSD, evaluating whether they may act as a useful biomarker to early identify and diagnose depression after stroke as well as to predict the recovery of PSD.

## Methods

### Search strategy

This systematic review was conducted in accord with the guidelines recommended by Cochrane Collaboration (52) and the checklist of Preferred Reporting Items for Systematic Reviews and Meta-Analyses (PRISMA). Relevant studies were searched by the following electronic databases from inception to 31 March 2022: Medline, Embase, Cochrane Library, Web of Science, PsycINFO database, China National Knowledge Infrastructure (CNKI), Wanfang Database, Chinese Scientific Journal Database (VIP database), and Chinese Biomedical Literature Database (CBM). The following keywords were searched: ((“urinary metabolite” [Title/Abstract]) OR (“urinary biomarkers” [Title/Abstract]) OR (“urine metabolomics” [Title/Abstract])) AND ((“post stroke depression” [Title/Abstract]) OR (“post stroke depressive disorder”[Title/Abstract]) OR (“depression after stroke” [Title/Abstract]) OR (“depressive disorder after stroke” [Title/Abstract])).

### Study selection

Inclusion criteria were as follows:

• Studies investigating urinary metabolite in patients with PSD;
• Studies assessing the depressive symptoms using diagnostic criteria or rating scales.

Exclusion criteria were as follows:

• Studies investigating other types of biomarkers from patients with PSD;
• Systematic reviews, meta-analyses, narrative reviews, book chapters, letters to the editor, case series, case reports, and animal studies.

Two researchers independently screened the titles and abstracts of searched articles and excluded unrelated studies, followed by the full-text review of the remaining articles by the same researchers. Studies published in English or Chinese were searched. Disagreements were resolved after a discussion with the third author.

### Data extraction

Data were extracted from all the included studies and recorded in an excel spreadsheet, including title, author, study design, participants’ characteristics, assessment of depression, and main findings. Any disagreement was resolved by a discussion with a third researcher.

### Quality assessment

Two independent researchers used the modified versions of the Newcastle-Ottawa Quality Assessment Scale to assess the quality of included studies (53). The selection, the main outcomes and comparability of the studies were investigated by the scale. A score of 7 or above indicated a good quality. A score of 5–6 was considered a satisfactory quality. Scores less than 5 suggested unsatisfactory studies. With respect to selection, studies were considered to have a low, medium, or high risk of bias if they scored 3, 1–2, or 0 point, respectively. In terms of comparability, studies were considered to have a low, medium, or high risk of bias if they scored 2, 1, or 0 point, respectively. Referring to the outcome, studies were considered to have a low, medium, or high risk of bias if they scored 3, 2, or 1 point, respectively. The disagreement was resolved by a third researcher.

## Results

**FIGURE 1:**
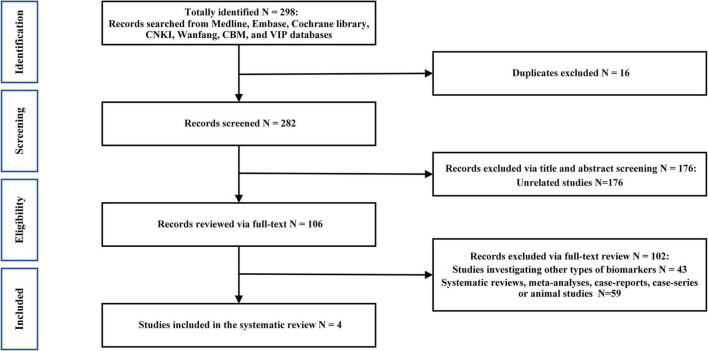
PRISMA flow diagram.

The initial literature search identified 301 results, of which only 4 were included in our analysis according to the inclusion criteria (Figure 1). The main characteristics of the included studies are summarized in Table 1. None of the studies mentioned that the recruited patients received any medication. Participants with alcohol abuse or illicit drug use were excluded in all the included studies.

**TABLE 1:** Characteristics of included studies.

Author	Study design	Sample size (N)	Age (Y)	Gender (M/F)	Stroke diagnosis	Depression assessment	Main findings
Zhang et al. (51)	Cross-Sectional Studies	**Training set** HC:74 NSS:72 PSD:72 **Test set** HC:53 NSS:56 PSD:58	**Training set** HC: 61.6 ± 5.8 NSS: 61.3 ± 8.7 PSD: 62.0 ± 6.3 **Test set** HC: 61.3 ± 6.1 NSS: 62.4 ± 8.0 PSD: 62.1 ± 8.3	**Training set** HC: 32/42 NSS: 39/33 PSD: 31/41 **Test set** HC: 24/29 NSS: 21/35 PSD: 30/28	Previously diagnosed with unclear criteria	DSM-IV, HDRS	Six metabolites, glyceric acid, azelaic acid, 5-hydroxyhexanoic acid, pseudouridine, phenylalanine and tyrosine were defined as biomarkers. A combined panel of these six urinary metabolites could effectively tell the difference between PSD and non-PSD. This urinary biomarker panel was able to discriminate blinded test samples with an AUC of 0.954 (*n* = 58 PSD patients and *n* = 109 non-PSD subjects).

Xie at al. (50)	Cross-sectional studies	**Training set** NSS:59 PSD:58 **Test set** NSS:30 PSD:34	**Training set** NSS: 52.27 ± 5.25 PSD: 54.27 ± 4.19 **Test set** NSS: 51.77 ± 4.49 PSD: 54.14 ± 4.01	**Training set** NSS: 28/21 PSD: 25/23 **Test set** NSS: 16/14 PSD: 19/15	The criteria confirmed by the fourth national conference on cerebrovascular diseases	HDRS	Seven metabolites (hydroxylamine, palmitic acid, glyceric acid, myristic acid, lactic acid, azelaic acid and tyrosine) were selected as potential biomarkers in the diagnosis of PSD for middle-aged stroke patients.

Liang et al. (49)	Cross-sectional studies	**Training set** NSS:45 PSD:55 **Test set** NSS:38 PSD:46	**Training set** NSS: 59.55 ± 9.73 PSD: 60.83 ± 8.27 **Test set** NSS: 58.39 ± 9.3 PSD: 61.36 ± 7.87	**Training set** NSS: 20/25 PSD: 27/28 **Test set** NSS: 17/21 PSD: 21/25	The criteria confirmed by the fourth national Conference on cerebrovascular diseases	HDRS	A panel consisting of malic acid, pseudouridine, hypoxanthine, fructose, 3, 4-dihydroxybutyric acid and inositol was identified, which could effectively discriminate PSD from non-depressed stroke survivors of T2DM patients. The galactose metabolism was significantly affected in T2DM patients with PSD.

Chen et al. (48)	Cross-sectional studies	**Training set** HC:44 NSS:86 PSD:82 **Test set** HC:34 NSS:36 PSD:42	**Training set** HC: 65.20 ± 3.71 NSS: 66.88 ± 4.84 PSD: 67.22 ± 4.69 **Test set** HC: 65.41 ± 2.74 NSS: 67.19 ± 5.42 PSD: 66.17 ± 4.34	**Training set** HC: 18/26 NSS: 26/40 PSD: 39/43 **Test set** HC: 17/17 NSS: 15/21 PSD: 21/21	The criteria confirmed by the fourth national conference on cerebrovascular diseases	DSM-IV, HDRS	The phenylalanine metabolism, tyrosine, phenylalanine and tryptophan biosynthesis, galactose metabolism were found to be significantly changed in elderly PSD subjects. The phenylalanine was significantly negatively correlated with depressive symptoms and age. A biomarker panel consisting of tyrosine, 3-hydroxyphenylacetic acid, phenylalanine, palmitic acid, sucrose, glyceric acid, α-aminobutyric acid and azelaic acid was identified.

DSM-IV, Diagnostic and Statistical Manual of Mental Disorders-IV; F, female; HC, healthy controls; HDRS, Hamilton Depression Rating Scale; M, male; N, number; NSS, non-depressed stroke survivors; PSD, post stroke depression; T2DM, type 2 diabetes mellitus; Y, year.

Table 2 presents the results of the quality assessment using the modified versions of the Newcastle-Ottawa Quality Assessment Scale. All the included studies were of good quality.

**TABLE 2:** Quality of the included studies.

Author	Selection				Comparability	Outcome		Overall
	Representativeness of the sample	Sample size	Non-respondents	Ascertainment of depression	Based on design and analysis	Assessment of the outcome	Statistical test	
Zhang et al. (51)	*	/	/	**	*	**	*	7
Xie et al. (50)	*	/	/	**	*	**	*	7
Liang et al. (49)	*	/	/	**	*	**	*	7
Chen et al. (48)	*	/	/	**	*	**	*	7

/, 0 point; *, 1 point; **, 2 points.

Xie et al.’s study (50) included 89 stroke survivors without depression and 92 patients with PSD. A total of 12 differential urinary metabolites were identified between patients with PSD and non-depressed stroke survivors, including hydroxylamine, palmitic acid, glucose, myristic acid, fructose, lactic acid, glyceric acid, azelaic acid, pyroglutamic acid, α-aminobutyric acid, uric acid, and tyrosine. Moreover, seven differential urinary metabolites in the panel were found to effectively disclose the difference between patients with PSD and non-depressed stroke survivors. The panel included hydroxylamine, myristic acid, palmitic acid, lactic acid, glyceric acid, azelaic acid, and tyrosine, demonstrating the differentiation of PSD. Interestingly, this study figured out four metabolic pathways, which included tyrosine, phenylalanine and tryptophan biosynthesis, glycerolipid metabolism, fatty acid biosynthesis, starch, and sucrose metabolism.

Liang et al.’s study (49) included 101 patients with PSD and 83 non-depressed patients after stroke. All the recruited patients were also diagnosed with type 2 diabetes mellitus (T2DM). The research identified 23 differential metabolites to discriminate the two groups. Patients in the PSD group were characterized by higher levels of sucrose, inositol, citric acid, lactic acid, methylsuccinic acid, vanillic acid, sorbitol, 2-methyl-3-hydroxybutyric acid, 3, 4-dihydroxybutyric acid, hydroxylamine, threitol, myristic acid, D-glucose, azelaic acid, palmitic acid, and fructose, along with lower levels of hypoxanthine, tyrosine, aminoethanol, malic acid, pseudouridine, indoxyl sulfate, and n-methylnicotinamide compared to non-depressed stroke survivors. Most of the differential metabolites were found to change significantly. Furthermore, the galactose metabolism was significantly affected in T2DM patients with PSD. The panel consisting of six differential metabolites including malic acid, pseudouridine, hypoxanthine, fructose, 3, 4-dihydroxybutyric acid, and inositol could effectively distinguish the two groups.

Zhang et al. (51) conducted a clinical study totally recruiting 130 patients with PSD, 128 non-depressed stroke survivors, and 127 HC candidates (28). A total of 17 differential urinary metabolites were identified to discriminate PSD from non-PSD. Higher levels of glyceric acid, azelaic acid, 5-hydroxyhexanoic acid, pseudouridine sucrose, lactic acid, and palmitic acid, along with lower levels of hippuric acid, tyrosine, phenylalanine, 3-hydroxyisobutyric acid, indoxyl sulfate, b-aminoisobutyric acid, leucine, hypoxanthine, ribose, and pyroglutamic acid were found in the PSD group compared with the non-PSD group. It was found that 6 metabolites (glyceric acid, azelaic acid, 5-hydroxyhexanoic acid, pseudouridine, phenylalanine, and tyrosine) significantly differentiated between PSD and non-PSD. The urinary biomarker panel could distinguish 72 patients with PSD from 146 non-PSD subjects.

Interestingly, Chen et al. (48) conducted a study investigating the elderly population including 124 patients with PSD, 78 HCs, and 122 non-depressed stroke survivors. Compared to elderly non-depressed subjects, the elderly subjects with PSD were characterized by higher levels of azelaic acid, palmitic acid, sucrose, α-aminobutyric acid, glyceric acid, fructose, and lactic acid, with lower levels of sorbitol, indoxyl sulfate, 3-hydroxyisobutyric acid, phenylalanine, tyrosine, and 3-hydroxyphenylacetic acid. Of these, 12 differential urinary metabolites remained significantly changed. The panel consisting of 8 differential urinary metabolites was the most significant deviations between elderly patients with PSD and elderly non-depressed subjects, including tyrosine, phenylalanine, 3-hydroxyphenylacetic acid, sucrose, palmitic acid, glyceric acid, α-aminobutyric acid, and azelaic acid. A significantly negative correlation was found between phenylalanine and age. Significantly negative correlations were also found between BMI and five differential urinary metabolites (sorbitol, tyrosine, phenylalanine, 3-hydroxyisobutyric acid, and azelaic acid). Furthermore, Hamilton Depression Rating Scale (HDRS) score and three differential urinary metabolites (3-hydroxyisobutyric acid, phenylalanine, and sucrose) were negatively correlated. In elderly subjects with PSD, three metabolic pathways, namely, phenylalanine metabolism, tyrosine, phenylalanine and tryptophan biosynthesis, and galactose metabolism were affected significantly.

According to all the included studies, the metabolites in the urine samples were profiled by a GC-MS-based metabolomics platform; the identification of differential urinary metabolites was analyzed by corresponding partial least-squares discriminant analysis (PLS-DA) loading plots. In addition, a simplified metabolite biomarker panel consisting of significantly different urinary metabolites was obtained to distinguish subjects with PSD from non-depressed stroke survivors *via* step-wise logistic-regression analysis. All of these are summarized in Table 3. Azelaic acid, lactic acid, palmitic acid, and tyrosine were identified in all the included studies. Most of the included studies (3 out of 4) identified these differential urinary metabolites including fructose, glyceric acid, indoxyl sulfate, and sucrose. Of all the significant deviations in the metabolite biomarker panel, glyceric acid, azelaic acid, phenylalanine, palmitic acid, tyrosine, and pseudouridine were found in at least 2 included studies, which indicated good potential for the differentiation of PSD. Moreover, of all the metabolic pathways detected, biosynthesis of phenylalanine, tyrosine, and tryptophan was significantly affected in 2 studies included.

**TABLE 3:** Summary of all the differential urinary metabolites identified in the included studies.

Urinary metabolites	Author of the included studies			
	Zhang et al. (51)	Xie at al. (50)	Liang et al. (49)	Chen et al. (48)
Aminoethanol	/	/	▲	/
Azelaic acid	▲ ¶	▲ ¶	▲	▲ ¶
Citric acid	/	/	▲	/
Fructose	/	▲	▲ ¶	▲
Glucose	/	▲	▲	/
Glyceric acid	▲ ¶	▲ ¶	/	▲ ¶
Hippuric acid	▲	/	/	/
Hydroxylamine	/	▲ ¶	▲	/
Hypoxanthine	▲	/	▲ ¶	/
Indoxyl sulfate	▲	/	▲	▲
Inositol	/	/	▲ ¶	/
Lactic acid	▲	▲ ¶	▲	▲
Leucine	▲	/	/	/
Malic acid	/	/	▲ ¶	/
Methylsuccinic acid	/	/	▲	/
Myristic acid	/	▲ ¶	▲	/
n-Methylnicotimide	/	/	▲	/
Palmitic acid	▲	▲ ¶	▲	▲ ¶
Phenylalanine	▲ ¶	/	/	▲ ¶
Pseudouridine	▲ ¶	/	▲ ¶	/
Pyroglutamic acid	▲	▲	/	/
Ribose	▲	/	/	/
Sorbitol	/	/	▲	▲
Sucrose	▲	/	▲	▲ ¶
Threitol	/	/	▲	/
Tyrosine	▲ ¶	▲ ¶	▲	▲ ¶
Uric acid	/	▲	/	/
Vanillic acid	/	/	▲	/
α-aminobutyric acid	/	▲	/	▲ ¶
β-aminoisobutyric acid	▲	/	/	/
2-methyl-3-hydroxybutyric acid	/	/	▲	/
3, 4-dihydroxybutyric acid	/	/	▲ ¶	/
3-hydroxyisobutyric acid	/	/	/	▲
3-hydroxyphenylacetic acid	/	/	/	▲ ¶
5-hydroxyhexanoic acid	▲ ¶	/	/	/

▲, Identified; /, Not identified; ¶, significant deviation.

## Discussion

The incidence rates of stroke vary between 10 and 200 per 10,000 individuals between the age of 55–85 years and over 85 years. Referring to the latest statistics of the American Heart Association, 700,000 strokes occur every year in the United States (54). Neuropsychiatric disorders associated with stroke include depression, apathy, anxiety disorder, psychosis, mania, cognitive impairment, anosognosia, and fatigue. It was found in Folstein et al.’s study (55) that the depressive disorder was significantly more common in patients with stroke compared to patients undergoing other physical dysfunctions. With a high prevalence rate, PSD is a potential predictor of poor functional outcomes associated with imitations in daily activities (56), poor rehabilitation outcomes, cognitive problems (57, 58), social isolation, suicidal ideation (59), and higher all-cause mortality (60). However, the diagnosis of PSD is inconsistent and challenging. The neurological symptoms associated with stroke such as aphasia, abulia, or dementia might hinder the detection of depressive symptoms (61). Furthermore, if the patient refuses to participate in the therapy, it is not easy to early detect the signs of PSD.

Precision medicine is a promising strategy to overcome obstacles in the detection and diagnosis of psychiatric disorders. The rapid development of biotechnologies has been able to realize the target of precision medicine (62). As one of the high-throughput biotechnologies, metabolomics could play important roles in health and disorders (63). The data’s accuracy and comprehensive dimensionality are responsible for the realization of precision psychiatry. Analytical techniques for metabolomics include MS, NMR, and electrochemical detection. NMR is good at identifying novel metabolite structures. More sensitive than NMR, MS-based methods have an advantage in mass analysis capabilities. GC or liquid chromatography (LC) is usually used together with MS-based methods in metabolomics studies. LC-MS is able to detect the compounds of low molecular weight with high sensitivity and selectivity. Characterized by high sensitivity and resolution, GC-MS is able to identify unknown compounds including both the less polar and volatile metabolites and some polar compounds (64). Of note, GC-MS-based techniques were used to precisely characterize the urinary metabolic profiling in all the included studies, which enhance the credibility and scientificity of the results.

The pathophysiology of depressive disorder is closely related to the IgM-mediated immune responses. Azelaic acid, a naturally occurring acid found in grains, has anti-inflammatory properties. Conjugated azelaic acid was directed against by increased antibody titers, indicating enhanced oxidation of fatty acids (65), which could be observed in depressive disorder. In addition, as one of the most common saturated fatty acids, palmitic acid has been oxidatively modified in depression. Higher IgM antibody levels were found to direct against palmitoyl in depressed patients compared with normal subjects.

Considering the fact that azelaic acid, lactic acid, palmitic acid, and tyrosine were identified in all the included studies, a suitable statistical analysis technique can be used in future studies to calculate the area under the curve (AUC) value of the four specific metabolites to act as a significant biomarker to diagnose PSD. Lactic acid is an alpha-hydroxy acid (AHA) on account of the presence of a hydroxyl group adjacent to the carboxyl group. Lactate is the conjugate base of lactic acid. A few studies reported that lactate is metabolized by cerebral neurons of humans (66, 67). Glial cells could transform glucose into lactate, which was provided to the neurons (68, 69). As a result, extracellular fluid such as blood or cerebrospinal fluid is richer in lactate. However, studies found that both glial cells and neurons in the hippocampus of the brain decrease in depressed patients (70, 71), which could explain the identification of lower lactic acid in depressed subjects compared to those non-depressed subjects after stroke. Half of the included studies significantly identified the metabolic pathway of tyrosine, phenylalanine, and tryptophan. Closely involved in the context of depressive symptoms, indoleamine 2, 3-dioxygenase (IDO) is the crosstalk of the serotonergic system, the immune activation, and the kynurenic acid pathway. The breakdown of tryptophan which is the essential precursor of 5-HT is caused by IDO. Phenylalanine is the substrate for phenylalanine 4-hydroxylase (PAH) that forms another important amino acid called tyrosine. Tyrosine is converted into L-dopamine (L-DOPA) which is converted into dopamine (DA) in the tyrosine metabolic pathway. Furthermore, DA is converted into noradrenaline (NA) by DA hydroxylase. Numerous studies (72–83) confirmed that the dysfunction of monoamine neurotransmitters such as NE, DA, and 5-HT plays a crucial part in the pathogenesis of depressive disorder. Elevated DA, L-DOPA, and vanillylmandelic acid (VMA) levels were found in the hippocampus of CSDS-induced model mice (84, 85). The levels of VMA and L-DOPA were reported to be related to depressive behaviors, which is consistent with the results of all the included studies that a higher level of tyrosine was identified in the PSD group than the non-depressed group. In contrast, patients with depression had reduced metabolites level of DA in the CSF (86) and in the hypothalamus of depression model mouse (87), results of which were consistent with the monoamine hypothesis. In summary, the different levels of metabolites might present the switch between depression and remission.

However, with regard to the plasma metabolites identified in PSD compared with non-depressed stroke survivors, the results of three previous studies were not consistent. Ding et al. (88) reported that subjects with PSD had elevated levels of oleic acid, palmitic acid, linoleic acid, pyroglutamate, proline, rhamnose, and decreased level of oxalate. In Wang et al.’s study (89), phenylacetylglutamine, p-chlorophenylalanine, and DHA levels were higher, while palmitic acid, betaine (trimethylglycine), and MHPG-SO4 were lower in the PSD group. Hu et al.’s study (90) found that the plasma of subjects with PSD was associated with higher levels of 3-methylhistidine, 1-methylhistidine, LDL CH_3_-(CH_2_)_*n*_-, phenylalanine, and lower levels of tyrosine. The inconsistent results of these three studies might be related to different techniques to analyze the plasma sample. GC-MS analysis was used in Ding et al.’s study, Wang et al. applied LC-MS analysis, and the third study was based on a 1H NMR-based approach. Of note, the results of all the included studies in the systematic review were consistent due to the unified use of the GC-MS-based metabolomics method. In contrast, a GC-MS-based study (91) investigating fecal metabolites identified in PSD compared to HCs and non-depressed stroke survivors found that differential metabolites include amino acid metabolism (5-methoxytryptamine, l-kynurenine, glutamate, tyramine, cyanoalanine, maleic acid, and phenylacetic acid), lipid metabolism (lanosterol, squalene, lignoceric acid, and stigmasterol), carbohydrate metabolism (n-acetyl-d-mannosamine, arbutin, acetyl alanine, glutamate, and sucrose-6-phosphate), and nucleotide metabolism (cytosine), inconsistent with the results of included GC-MS-based research studying urinary metabolites. Therefore, the same metabonomic approach applied to analyze the same body fluid or tissue might be the reasonable strategy to precisely characterize the metabolic profiling as the diagnostic biomarker of PSD.

Nonetheless, it should be noted that only four related studies with a small sample size were included in the systematic review due to that very few studies have investigated this subject to date. Further studies investigating subgroups of different gender and age could better figure out biological differences among patients with PSD to guide more personalized treatment strategies. More studies of large sample size are also needed to investigate the essential mechanism through how urinary metabolites may interact in PSD. Furthermore, more randomized controlled trial (RCT) studies are needed to clarify the metabolomics-related role of antidepressants in alleviating depression after stroke.

## Conclusion

The systematic review provided potential evidence that the GC-MS-based urinary metabolomics approach might be a useful diagnostic tool for PSD. Differential urinary metabolites significantly identified in those depressed compared to those non-depressed after stroke could be used as a precise biomarker for the diagnosis and prognosis of PSD.

## Author contributions

WC and W-DS designed the study. WC, X-FWa, X-FWe, J-RZ, CH, WM, and W-DS performed the study. WC drafted the manuscript. All authors have read and approved the final manuscript.

## References

[1] Whyte EM Mulsant BH. Post stroke depression: Epidemiology, pathophysiology, and biological treatment. *Biol Psychiatry.* (2002) 52:253–64. 10.1016/S0006-3223(02)01424-5 12182931

[2] Camargos QM Silva BC Silva DG Toscano ECB Oliveira BDS Bellozi PMQ Minocycline treatment prevents depression and anxiety-like behaviors and promotes neuroprotection after experimental ischemic stroke. *Brain Res Bull.* (2020) 155:1–10. 10.1016/j.brainresbull.2019.11.009 31756420

[3] Lanctot KL Lindsay MP Smith EE Sahlas DJ Foley N Gubitz G Canadian stroke best practice recommendations: Mood, cognition and fatigue following stroke, 6th edition update 2019. *Int J Stroke Off J Int Stroke Soc.* (2020) 15:668–88. 10.1177/1747493019847334 31221036

[4] Liu X Wang B Wang X Tian M Wang X Zhang Y. Elevated plasma high-sensitivity C-reactive protein at admission predicts the occurrence of post-stroke fatigue at 6 months after ischaemic stroke. *Eur J Neurol.* (2020) 27:2022–30. 10.1111/ene.14430 32633437

[5] Carnes-Vendrell A Deus J Molina-Seguin J Pifarre J Purroy F. Depression and apathy after transient ischemic attack or minor stroke: Prevalence, evolution and predictors. *Sci Rep.* (2019) 9:16248. 10.1038/s41598-019-52721-5 31700058 PMC6838079

[6] Baylan S Griffiths S Grant N Broomfield NM Evans JJ Gardani M. Incidence and prevalence of post-stroke insomnia: A systematic review and meta-analysis. *Sleep Med Rev.* (2020) 49:101222. 10.1016/j.smrv.2019.101222 31739180

[7] Devine MJ Bentley P Jones B Hotton G Greenwood RJ Jenkins IH The role of the right inferior frontal gyrus in the pathogenesis of post-stroke psychosis. *J Neurol.* (2014) 261:600–3. 10.1007/s00415-014-7242-x 24449063 PMC3948509

[8] Koreki A Takahata K Tabuchi H Kato M. Increased left anterior insular and inferior prefrontal activity in post-stroke mania. *BMC Neurol.* (2012) 12:68. 10.1186/1471-2377-12-68 22866872 PMC3482600

[9] Mijajlovic MD Pavlovic A Brainin M Heiss WD Quinn TJ Ihle-Hansen HB Post-stroke dementia - a comprehensive review. *BMC Med.* (2017) 15:11. 10.1186/s12916-017-0779-7 28095900 PMC5241961

[10] Sachdev PS. Post-stroke cognitive impairment, depression and apathy: Untangling the relationship. *Am J Geriatr Psychiatry.* (2018) 26:301–3. 10.1016/j.jagp.2017.12.002 29325929

[11] Biran I Chatterjee A. Depression with anosognosia following a left subcortical stroke. *Clin Neurol Neurosurg.* (2003) 105:99–101. 10.1016/S0303-8467(02)00113-0 12691800

[12] Hackett ML Pickles K. Part I: Frequency of depression after stroke: An updated systematic review and meta-analysis of observational studies. *Int J Stroke Off J Int Stroke Soc.* (2014) 9:1017–25. 10.1111/ijs.12357 25117911

[13] Aben I Denollet J Lousberg R Verhey F Wojciechowski F Honig A. Personality and vulnerability to depression in stroke patients: A 1-year prospective follow-up study. *Stroke.* (2002) 33:2391–5. 10.1161/01.STR.0000029826.41672.2E 12364726

[14] Andersen G Vestergaard K Riis J Lauritzen L. Incidence of post-stroke depression during the first year in a large unselected stroke population determined using a valid standardized rating scale. *Acta Psychiatrica Scandinavica.* (1994) 90:190–5. 10.1111/j.1600-0447.1994.tb01576.x 7810342

[15] Appelros P Viitanen M. Prevalence and predictors of depression at one year in a Swedish population-based cohort with first-ever stroke. *J Stroke Cerebrovasc Dis Off J Natl Stroke Assoc.* (2004) 13:52–7. 10.1016/j.jstrokecerebrovasdis.2004.02.005 17903950

[16] Ayerbe L Ayis S Rudd AG Heuschmann PU Wolfe CD. Natural history, predictors, and associations of depression 5 years after stroke: The South London Stroke Register. *Stroke.* (2011) 42:1907–11. 10.1161/STROKEAHA.110.605808 21566241

[17] Grabowska-Fudala B Jaracz K Gorna K Miechowicz I Wojtasz I Jaracz J Depressive symptoms in stroke patients treated and -treated with intravenous thrombolytic therapy: A 1-year follow-up study. *J Neurol.* (2018) 265:1891–9. 10.1007/s00415-018-8938-0 29916129 PMC6060771

[18] Jorgensen TS Wium-Andersen IK Wium-Andersen MK Jorgensen MB Prescott E Maartensson S Incidence of depression after stroke, and associated risk factors and mortality outcomes, in a large cohort of danish patients. *JAMA Psychiatry.* (2016) 73:1032–40. 10.1001/jamapsychiatry.2016.1932 27603000

[19] Liman TG Heuschmann PU Endres M Floel A Schwab S Kolominsky-Rabas PL. Impact of low mini-mental status on health outcome up to 5 years after stroke: The Erlangen Stroke Project. *J Neurol.* (2012) 259:1125–30. 10.1007/s00415-011-6312-6 22109634

[20] Sienkiewicz-Jarosz H Milewska D Bochynska A Chelmniak A Dworek N Kasprzyk K Predictors of depressive symptoms in patients with stroke - a three-month follow-up. *Neurol Neurochir Pol.* (2010) 44:13–20. 10.1016/S0028-3843(14)60402-3 20358481

[21] Sturm JW Donnan GA Dewey HM Macdonell RA Gilligan AK Thrift AG. Determinants of handicap after stroke: The North East Melbourne Stroke Incidence Study (NEMESIS). *Stroke.* (2004) 35:715–20. 10.1161/01.STR.0000117573.19022.66 14963272

[22] Tsai CS Wu CL Hung TH Chou SY Su JA. Incidence and risk factors of poststroke depression in patients with acute ischemic stroke: A 1-year prospective study in Taiwan. *Biomed J.* (2016) 39:195–200. 10.1016/j.bj.2015.10.004 27621121 PMC6140301

[23] Zhang WN Pan YH Wang XY Zhao Y. A prospective study of the incidence and correlated factors of post-stroke depression in China. *PLoS One.* (2013) 8:e78981. 10.1371/journal.pone.0078981 24260141 PMC3832506

[24] Ayerbe L Ayis S Wolfe CD Rudd AG. Natural history, predictors and outcomes of depression after stroke: Systematic review and meta-analysis. *Br J Psychiatry J Ment Sci.* (2013) 202:14–21. 10.1192/bjp.bp.111.107664 23284148

[25] Hackett ML Anderson CS. Predictors of depression after stroke: A systematic review of observational studies. *Stroke.* (2005) 36:2296–301. 10.1161/01.STR.0000183622.75135.a4 16179565

[26] Pan A Sun Q Okereke OI Rexrode KM Hu FB. Depression and risk of stroke morbidity and mortality: A meta-analysis and systematic review. *JAMA.* (2011) 306:1241–9. 10.1001/jama.2011.1282 21934057 PMC3242806

[27] Taylor-Rowan M Momoh O Ayerbe L Evans JJ Stott DJ Quinn TJ. Prevalence of pre-stroke depression and its association with post-stroke depression: A systematic review and meta-analysis. *Psychol Med.* (2019) 49:685–96. 10.1017/S0033291718002003 30107864

[28] Liu Q Wang X Wang Y Wang C Zhao X Liu L Association between marriage and outcomes in patients with acute ischemic stroke. *J Neurol.* (2018) 265:942–8. 10.1007/s00415-018-8793-z 29464375 PMC5878185

[29] Northcott S Moss B Harrison K Hilari K. A systematic review of the impact of stroke on social support and social networks: Associated factors and patterns of change. *Clin Rehabil.* (2016) 30:811–31. 10.1177/0269215515602136 26330297

[30] Shi Y Yang D Zeng Y Wu W. Risk factors for post-stroke depression: A meta-analysis. *Front Aging Neurosci.* (2017) 9:218. 10.3389/fnagi.2017.00218 28744213 PMC5504146

[31] Backhouse EV McHutchison CA Cvoro V Shenkin SD Wardlaw JM. Cognitive ability, education and socioeconomic status in childhood and risk of post-stroke depression in later life: A systematic review and meta-analysis. *PLoS One.* (2018) 13:e0200525. 10.1371/journal.pone.0200525 30011299 PMC6047794

[32] Barca ML Eldholm RS Persson K Bjorklof GH Borza T Telenius E Cortisol levels among older people with and without depression and dementia. *Int Psychogeriatr.* (2019) 31:597–601. 10.1017/S1041610218001199 30556798

[33] Doolin K Farrell C Tozzi L Harkin A Frodl T O’Keane V. Diurnal hypothalamic-pituitary-adrenal axis measures and inflammatory marker correlates in major depressive disorder. *Int J Mol Sci.* (2017) 18:2226. 10.3390/ijms18102226 29064428 PMC5666905

[34] Danese A Pariante CM Caspi A Taylor A Poulton R. Childhood maltreatment predicts adult inflammation in a life-course study. *Proc Natl Acad Sci U S A.* (2007) 104:1319–24. 10.1073/pnas.0610362104 17229839 PMC1783123

[35] Setiawan E Wilson AA Mizrahi R Rusjan PM Miler L Rajkowska G Role of translocator protein density, a marker of neuroinflammation, in the brain during major depressive episodes. *JAMA Psychiatry.* (2015) 72:268–75. 10.1001/jamapsychiatry.2014.2427 25629589 PMC4836849

[36] Villa RF Ferrari F Moretti A. Post-stroke depression: Mechanisms and pharmacological treatment. *Pharmacol Ther.* (2018) 184:131–44. 10.1016/j.pharmthera.2017.11.005 29128343

[37] Chen HH Zhang N Li WY Fang MR Zhang H Fang YS Overexpression of brain-derived neurotrophic factor in the hippocampus protects against post-stroke depression. *Neural Regen Res.* (2015) 10:1427–32. 10.4103/1673-5374.165510 26604903 PMC4625508

[38] Li J Zhao YD Zeng JW Chen XY Wang RD Cheng SY. Serum Brain-derived neurotrophic factor levels in post-stroke depression. *J Affect Disord.* (2014) 168:373–9. 10.1016/j.jad.2014.07.011 25106034

[39] Zhang E Liao P. Brain-derived neurotrophic factor and post-stroke depression. *J Neurosci Res.* (2020) 98:537–48. 10.1002/jnr.24510 31385340

[40] Goldberg DP Prisciandaro JJ Williams P. The primary health care version of ICD-11: The detection of common mental disorders in general medical settings. *Gen Hosp Psychiatry.* (2012) 34:665–70. 10.1016/j.genhosppsych.2012.06.006 22832134

[41] Clark LA Cuthbert B Lewis-Fernandez R Narrow WE Reed GM. Three approaches to understanding and classifying mental disorder: ICD-11, DSM-5, and the National Institute of Mental Health’s Research Domain Criteria (RDoC). *Psychol Sci Public Interest.* (2017) 18:72–145. 10.1177/1529100617727266 29211974

[42] Kessler RC. Psychiatric epidemiology: Challenges and opportunities. *Int Rev Psychiatry.* (2007) 19:509–21. 10.1080/09540260701564914 17896231 PMC2140947

[43] Howland RH. Sequenced Treatment Alternatives to Relieve Depression (STAR*D). Part 2: Study outcomes. *J Psychosoc Nurs Ment Health Serv.* (2008) 46:21–4. 10.3928/02793695-20081001-05 18935932

[44] Beger RD Dunn W Schmidt MA Gross SS Kirwan JA Cascante M Metabolomics enables precision medicine: “A White Paper, Community Perspective”. *Metabolomics.* (2016) 12:149. 10.1007/s11306-016-1094-6 27642271 PMC5009152

[45] Chen JJ Zhou CJ Zheng P Cheng K Wang HY Li J Differential urinary metabolites related with the severity of major depressive disorder. *Behav Brain Res.* (2017) 332:280–7. 10.1016/j.bbr.2017.06.012 28624318

[46] Gupta M Neavin D Liu D Biernacka J Hall-Flavin D Bobo WV TSPAN5, ERICH3 and selective serotonin reuptake inhibitors in major depressive disorder: Pharmacometabolomics-informed pharmacogenomics. *Mol Psychiatry.* (2016) 21:1717–25. 10.1038/mp.2016.6 26903268 PMC5003027

[47] Zhu H Bogdanov MB Boyle SH Matson W Sharma S Matson S Pharmacometabolomics of response to sertraline and to placebo in major depressive disorder - possible role for methoxyindole pathway. *PLoS One.* (2013) 8:e68283. 10.1371/journal.pone.0068283 23874572 PMC3714282

[48] Chen J Lv YN Li XB Xiong JJ Liang HT Xie L Urinary metabolite signatures for predicting elderly stroke survivors with depression. *Neuropsychiatr Dis Treat.* (2021) 17:925–33. 10.2147/NDT.S299835 33790561 PMC8007561

[49] Liang ZH Jia YB Li ZR Li M Wang ML Yun YL Urinary biomarkers for diagnosing poststroke depression in patients with type 2 diabetes mellitus. *Diabetes Metab Syndr Obes.* (2019) 12:1379–86. 10.2147/DMSO.S215187 31496775 PMC6698178

[50] Xie J Han Y Hong Y Li WW Pei Q Zhou X Identification of potential metabolite markers for middle-aged patients with post-stroke depression using urine metabolomics. *Neuropsychiatr Dis Treat.* (2020) 16:2017–24. 10.2147/NDT.S271990 32922015 PMC7457842

[51] Zhang W Zhang XA. A novel urinary metabolite signature for non-invasive post-stroke depression diagnosis. *Cell Biochem Biophys.* (2015) 72:661–7. 10.1007/s12013-014-0472-9 27352185

[52] Cumpston M Li T Page MJ Chandler J Welch VA Higgins JP Updated guidance for trusted systematic reviews: A new edition of the Cochrane Handbook for Systematic Reviews of Interventions. *Cochrane Database Syst Rev.* (2019) 10:ED000142. 10.1002/14651858.ED000142 31643080 PMC10284251

[53] Wells GA Shea B O’Connell D Peterson J Welch V Losos M *The Newcastle-Ottawa Scale (NOS) for assessing the quality of nonrandomised studies in meta-analyses.* Ottawa: Ottawa General Hospital (2013).

[54] Mozaffarian D Benjamin EJ Go AS Arnett DK Blaha MJ Cushman M Heart disease and stroke statistics–2015 update: A report from the American Heart Association. *Circulation.* (2015) 131:e29–322. 25520374 10.1161/CIR.0000000000000152

[55] Folstein MF Maiberger R McHugh PR. Mood disorder as a specific complication of stroke. *J Neurol Neurosurg Psychiatry.* (1977) 40:1018–20. 10.1136/jnnp.40.10.1018 591971 PMC492887

[56] Pohjasvaara T Vataja R Leppavuori A Kaste M Erkinjuntti T. Depression is an independent predictor of poor long-term functional outcome post-stroke. *Eur J Neurol.* (2001) 8:315–9. 10.1046/j.1468-1331.2001.00182.x 11422427

[57] Chemerinski E Robinson RG Kosier JT. Improved recovery in activities of daily living associated with remission of poststroke depression. *Stroke.* (2001) 32:113–7. 10.1161/01.STR.32.1.113 11136924

[58] Serrano S Domingo J Rodriguez-Garcia E Castro MD del Ser T. Frequency of cognitive impairment without dementia in patients with stroke: A two-year follow-up study. *Stroke.* (2007) 38:105–10. 10.1161/01.STR.0000251804.13102.c0 17158334

[59] Bartoli F Pompili M Lillia N Crocamo C Salemi G Clerici M Rates and correlates of suicidal ideation among stroke survivors: A meta-analysis. *J Neurol Neurosurg Psychiatry.* (2017) 88:498–504. 10.1136/jnnp-2017-315660 28331011

[60] Cai W Mueller C Li YJ Shen WD Stewart R. Post stroke depression and risk of stroke recurrence and mortality: A systematic review and meta-analysis. *Ageing Res Rev.* (2019) 50:102–9. 10.1016/j.arr.2019.01.013 30711712

[61] Duncan PW Zorowitz R Bates B Choi JY Glasberg JJ Graham GD Management of Adult Stroke Rehabilitation Care: A clinical practice guideline. *Stroke.* (2005) 36:e100–43. 10.1161/01.STR.0000180861.54180.FF 16120836

[62] Collins FS Varmus H. A new initiative on precision medicine. *N Engl J Med.* (2015) 372:793–5. 10.1056/NEJMp1500523 25635347 PMC5101938

[63] Hasin Y Seldin M Lusis A. Multi-omics approaches to disease. *Genome Biol.* (2017) 18:83. 10.1186/s13059-017-1215-1 28476144 PMC5418815

[64] Beale DJ Pinu FR Kouremenos KA Poojary MM Narayana VK Boughton BA Review of recent developments in GC-MS approaches to metabolomics-based research. *Metabolomics.* (2018) 14:152. 10.1007/s11306-018-1449-2 30830421

[65] Michel G Bodet D Martinet Y Dabadie MP. Detection of the specific IgM and IgA circulating in sera of multiple sclerosis patients: Interest and perspectives. *Immuno Anal Biol Specialisee.* (2002) 17:302–10. 10.1016/S0923-2532(02)01214-0

[66] Zilberter Y Zilberter T Bregestovski P. Neuronal activity in vitro and the in vivo reality: The role of energy homeostasis. *Trends Pharmacol Sci.* (2010) 31:394–401. 10.1016/j.tips.2010.06.005 20633934

[67] Wyss MT Jolivet R Buck A Magistretti PJ Weber B. In vivo evidence for lactate as a neuronal energy source. *J Neurosci Off J Soc Neurosci.* (2011) 31:7477–85. 10.1523/JNEUROSCI.0415-11.2011 21593331 PMC6622597

[68] Gladden LB. Lactate metabolism: A new paradigm for the third millennium. *J Physiol.* (2004) 558(Pt 1):5–30. 10.1113/jphysiol.2003.058701 15131240 PMC1664920

[69] Pellerin L Bouzier-Sore AK Aubert A Serres S Merle M Costalat R Activity-dependent regulation of energy metabolism by astrocytes: An update. *Glia.* (2007) 55:1251–62. 10.1002/glia.20528 17659524

[70] Bras JP Guillot de Suduiraut I Zanoletti O Monari S Meijer M Grosse J Stress-induced depressive-like behavior in male rats is associated with microglial activation and inflammation dysregulation in the hippocampus in adulthood. *Brain Behav Immun.* (2022) 99:397–408. 10.1016/j.bbi.2021.10.018 34793941

[71] Li X Sun X Xie J Wan H. CircDYM ameliorates CUMS mice depressive-like behavior and inhibits hippocampal neurons injury via miR-497a-5p/NR3C1 axis. *Brain Res.* (2022) 1787:147911. 10.1016/j.brainres.2022.147911 35413277

[72] Meng P Li C Duan S Ji S Xu Y Mao Y Epigenetic mechanism of 5-HT/NE/DA triple reuptake inhibitor on adult depression susceptibility in early stress mice. *Front Pharmacol.* (2022) 13:848251. 10.3389/fphar.2022.848251 35370730 PMC8968447

[73] Chen KZ Chen S Ren JY Lin S Xiao MJ Cheng L Antidepressant effect of acidic polysaccharides from Poria and their regulation of neurotransmitters and NLRP3 pathway. *China J Chin Materia Med.* (2021) 46:5088–95. 10.19540/j.cnki.cjcmm.20210610.705 34738405

[74] Yang HL Li MM Zhou MF Xu HS Huan F Liu N Links between gut dysbiosis and neurotransmitter disturbance in chronic restraint stress-induced depressive behaviours: The role of inflammation. *Inflammation.* (2021) 44:2448–62. 10.1007/s10753-021-01514-y 34657991

[75] Jiang N Wang H Huang H Lv J Zeng G Wang Q The antidepressant-like effects of shen yuan in a chronic unpredictable mild stress rat model. *Front Psychiatry.* (2021) 12:622204. 10.3389/fpsyt.2021.622204 33584387 PMC7876232

[76] Jiang JL El Mansari M Blier P. Triple reuptake inhibition of serotonin, norepinephrine, and dopamine increases the tonic activation of alpha2-adrenoceptors in the rat hippocampus and dopamine levels in the nucleus accumbens. *Progr Neuro Psychopharmacol Biol Psychiatry.* (2020) 103:109987. 10.1016/j.pnpbp.2020.109987 32474007

[77] Legakis LP Karim-Nejad L Negus SS. Effects of repeated treatment with monoamine-transporter-inhibitor antidepressants on pain-related depression of intracranial self-stimulation in rats. *Psychopharmacology.* (2020) 237:2201–12. 10.1007/s00213-020-05530-y 32382785 PMC7308219

[78] Peng Y Su Y Jiang Y. Effect of the warming and tonifying kidney- yang recipe on monoamine neurotransmitters and pathological morphology of hippocampus tissue in depression model rats. *Technol Health Care.* (2020) 28(Suppl 1):237–44. 10.3233/THC-209024 32364156 PMC7369113

[79] Li KD Yan K Wang QS Tian JS Xu D Zhang WY Antidepressant-like effects of dietary gardenia blue pigment derived from genipin and tyrosine. *Food Funct.* (2019) 10:4533–45. 10.1039/C9FO00480G 31264676

[80] Miao M Peng M Chen H Liu B. Effects of Baihe Dihuang powder on chronic stress depression rat models. *Saudi J Biol Sci.* (2019) 26:582–8. 10.1016/j.sjbs.2018.12.002 30899175 PMC6408709

[81] Liu Y Zhao J Guo W. Emotional roles of mono-aminergic neurotransmitters in major depressive disorder and anxiety disorders. *Front Psychol.* (2018) 9:2201. 10.3389/fpsyg.2018.02201 30524332 PMC6262356

[82] Ebrahimzadeh M El Mansari M Blier P. Synergistic effect of aripiprazole and escitalopram in increasing serotonin but not norepinephrine neurotransmission in the rat hippocampus. *Neuropharmacology.* (2019) 146:12–8. 10.1016/j.neuropharm.2018.11.006 30414871

[83] Dou M Gong A Liang H Wang Q Wu Y Ma A Improvement of symptoms in a rat model of depression through combined zinc and folic acid administration via up-regulation of the Trk B and NMDA. *Neurosci Lett.* (2018) 683:196–201. 10.1016/j.neulet.2018.07.036 30056106

[84] Wang W Guo H Zhang SX Li J Cheng K Bai SJ Targeted metabolomic pathway analysis and validation revealed glutamatergic disorder in the prefrontal cortex among the chronic social defeat stress mice model of depression. *J Proteome Res.* (2016) 15:3784–92. 10.1021/acs.jproteome.6b00577 27599184

[85] Xu K He Y Chen X Tian Y Cheng K Zhang L Validation of the targeted metabolomic pathway in the hippocampus and comparative analysis with the prefrontal cortex of social defeat model mice. *J Neurochem.* (2019) 149:799–810. 10.1111/jnc.14641 30520040

[86] Kaddurah-Daouk R Yuan P Boyle SH Matson W Wang Z Zeng ZB Cerebrospinal fluid metabolome in mood disorders-remission state has a unique metabolic profile. *Sci Rep.* (2012) 2:667. 10.1038/srep00667 22993692 PMC3446657

[87] Wu Y Wei Z Li Y Wei C Li Y Cheng P Perturbation of ephrin receptor signaling and glutamatergic transmission in the hypothalamus in depression using proteomics integrated with metabolomics. *Front Neurosci.* (2019) 13:1359. 10.3389/fnins.2019.01359 31920518 PMC6928102

[88] Ding X Liu R Li W Ni H Liu Y Wu D A metabonomic investigation on the biochemical perturbation in post-stroke patients with depressive disorder (PSD). *Metab Brain Dis.* (2016) 31:279–87. 10.1007/s11011-015-9748-z 26537495

[89] Wang M Gui X Wu L Tian S Wang H Xie L Amino acid metabolism, lipid metabolism, and oxidative stress are associated with post-stroke depression: A metabonomics study. *BMC Neurol.* (2020) 20:250. 10.1186/s12883-020-01780-7 32563250 PMC7305607

[90] Hu Z Fan S Liu M Zhong J Cao D Zheng P Objective diagnosis of post-stroke depression using NMR-based plasma metabonomics. *Neuropsychiatr Dis Treat.* (2019) 15:867–81. 10.2147/NDT.S192307 31118636 PMC6498396

[91] Jiang W Gong L Liu F Ren Y Mu J. Alteration of gut microbiome and correlated lipid metabolism in post-stroke depression. *Front Cell Infect Microbiol.* (2021) 11:663967. 10.3389/fcimb.2021.663967 33968807 PMC8100602

